# Supplementary material for: Cerebrospinal fluid levels of neuron-specific enolase predict the severity of brain damage in newborns with neonatal hypoxic-ischemic encephalopathy treated with hypothermia
Source: PLoS One. 2020 Jun 1;15(6):e0234082. doi: 10.1371/journal.pone.0234082 (PMC7263594; doi:10.1371/journal.pone.0234082)
Supplement: S1 Table — The three infants that did not undergo hypothermia treatment had severe HIE and were not included in this analysis (NSE values were 166, 281, and 2855 ng/ml). CSF-NSE values are expressed as median (interquartile range). Mann-Whitney U test was used to analyze the differences in CSF-NSE levels between groups. P value of <0.05 was considered as indicating statistical significance. aEEG: amplitude integrated electroencephalogram; BGP: background pattern; BSID-III: Bayley Scales of Infant and Toddler Development Third Edition; CP: cerebral palsy; MRI: magnetic resonance imaging; TH: therapeutic hypothermia. aOutcome in 28/29 surviving infants. bAdverse outcome: death or cerebral palsy and/or BSID-III score <85. (DOCX) [file pone.0234082.s001.docx]

**S1 Table. Cerebrospinal fluid levels of neuron-specific enolase of the 40 cooled infants with HIE according to their neurological findings and outcomes.**

| **Variables** | **Yes** | | **No** | | **P value** |
| --- | --- | --- | --- | --- | --- |
|  | **n** | **CSF-NSE (ng/ml)** | **n** | **CSF-NSE (ng/ml)** |  |
| Moderate or severe encephalopathy | 29 | 110.0 (52.5,251.5) | 11 | 26 (18, 33) | <0.001 |
| Electrical seizures during TH | 22 | 121 (70,267.3) | 18 | 27 (20.8,36.3) | <0.001 |
| Abnormal aEEG BGP in first 6 hours of life | 19 | 122 (77,299) | 19 | 33 (25, 59) | <0.001 |
| Abnormal aEEG BGP in first 80 hours of life | 21 | 144 (78.5,283.5) | 19 | 32 (23; 50) | <0.001 |
| Moderate-severe injury (MRI) | 18 | 121 (66.3, 267.3) | 18 | 28.5 (20.8, 36.3) | <0.001 |
| Global injury pattern (MRI) | 15 | 144 (80,268) | 21 | 29 (22, 46) | <0.001 |
| Cerebral palsy | 4 | 236.5 (92.5,291.3) | 23 | 33 (23; 59) | 0.006 |
| CP and/or BSID-III Score < 85^a^ | 7 | 110 (55,268) | 20 | 28.5 (21.5, 37) | <0.001 |
| CP and/or BSID-III Score < 70^a^ | 4 | 236.5 (62.5,291.3) | 23 | 33 (23, 59) | 0.006 |
| Adverse outcome^b^ | 19 | 201 (110,299) | 20 | 29 (21.5; 36.8) | <0.001 |
| Death | 12 | 218.5 (12.5,452) | 28 | 33.5 (25,70) | <0.001 |
