# Supplementary material for: Cerebrospinal fluid levels of neuron-specific enolase predict the severity of brain damage in newborns with neonatal hypoxic-ischemic encephalopathy treated with hypothermia
Source: PLoS One. 2020 Jun 1;15(6):e0234082. doi: 10.1371/journal.pone.0234082 (PMC7263594; doi:10.1371/journal.pone.0234082)
Supplement: S2 Table — The three infants that did not undergo hypothermia treatment had severe HIE and were not included in this analysis (NSE values were 166, 281 and 2855 ng/ml). aEEG: amplitude integrated electroencephalogram; BGP: background pattern; BSID-III: Bayley Scales of Infant and Toddler Development Third Edition; CP: cerebral palsy; MRI: magnetic resonance imaging; PPV: positive predictive value; NPV: negative predictive value; ROC: Receiver operating curve; TH: therapeutic hypothermia. aOutcome in 28/29 surviving infants. bAdverse outcome: death or cerebral palsy and/or BSID-III score <85. CSF-NSE values are expressed in ng/ml. (DOCX) [file pone.0234082.s002.docx]

**S2 Table . Receiver operating characteristic curve analysis of cerebrospinal fluid levels of neuron-specific enolase and neurological findings of the 40 cooled infants.**

| Variable | N | Cutoff-point | Area under the ROC (95%CI) | Sensitivity (95%CI) | Specificity (95%CI) | PPV (95%CI) | NPV (95%CI) |
| --- | --- | --- | --- | --- | --- | --- | --- |
| Moderate-to-severe HIE | 29 | 50 | 0.90 (0.81,0.99) | 0.79 (0.60,0.92) | 1.00 (0.71,-) | 1.00 (0.84,1.00) | 0.65 (0.42,-) |
| Severe HIE | 17 | 110 | 0.98 (0.94,1.00) | 0.82 (0.64,0.99) | 1.00 (0.85,-) | 1.00 (0.79,1.00) | 0.92 (0.73,-) |
| Electrical seizures during TH | 22 | 50 | 0.89 (0.78,1.00) | 0.91 (0.71,0.99) | 0.83 (0.59,0.96) | 0.87 (0.65,0.98) | 0.88 (0.65, 0.98) |
| Abnormal aEEG BGP in first 6 hours of life | 19 | 77 | 0.86 (0.74, 0.99) | 0.79 (0.54,0.53) | 0.89 (0.67,0.99) | 0.88 (0.64,0.67) | 0.81 (0.58,0.97) |
| Abnormal aEEG BGP in first 80 hours of life | 21 | 77 | 0.91 (0.81,1.00) | 0.81 (0.58,0.95) | 0.95 (0.74,0.99) | 0.94 (0.73,0.99) | 0.81 (0.59,0.99) |
| Moderate-severe injury (MRI) | 18 | 80 | 0.92 (0.83,1.00) | 0.72 (0.47,0.90) | 1.00 (0.81,-) | 1.00 (0.76,1.00) | 0.78 (0.55,-) |
| Global injury pattern (MRI) | 15 | 80 | 0.94 (0.87,1.00) | 0.80 (0.52,0.96) | 0.95 (0.76,0.99) | 0.92 (0.66,0.99) | 0.87 (0.64,0.99) |
| Cerebral palsy | 4 | 205 | 0.94 (0.80,1.00) | 0.65 (0.19,0.99) | 1.00 (0.85,1.00) | 1.00 (0.43,1.00) | 0.96 (0.65,-) |
| CP and/or BSID-III Score < 85^a^ | 7 | 50 | 0.91 (0.80,1.00) | 1.00 (0.59,-) | 0.80 (0.56,0.94) | 0.63 (0.36,-) | 1.00 (0.77,1.00) |
| CP and/or BSID-III Score < 70^a^ | 4 | 205 | 0.94 (0.80,1.00) | 0.65 (0.19,0.99) | 1.00 (0.85,1.00) | 1.00 (0.43,1.00) | 0.96 (0.65,-) |
| Adverse outcome^b^ | 19 | 108 | 0.97 (0.92,1.00) | 0.84 (0.60,0.97) | 1.00 (0.83,-) | 1.00 (0.80,1.00) | 0.87 (0.66,-) |
| Death | 12 | 108 | 0.93 (0.85,1.00) | 1.00 (0.74,-) | 0.86 (0.67,0.96) | 0.75 (0.51,-) | 1.00 (0.85,1.00) |
